# Stearoyl-CoA-desaturase-1 regulates gastric cancer stem-like properties and promotes tumour metastasis via Hippo/YAP pathway

**DOI:** 10.1038/s41416-020-0827-5

**Published:** 2020-04-30

**Authors:** Yunhe Gao, Jiyang Li, Hongqing Xi, Jianxin Cui, Kecheng Zhang, Jiabing Zhang, Yanmei Zhang, Wei Xu, Wenquan Liang, Ziwei Zhuang, Pengpeng Wang, Zhi Qiao, Bo Wei, Lin Chen

**Affiliations:** 1 Department of General Surgery, Chinese PLA General Hospital, Beijing, China; 2 Medical School of Chinese PLA General Hospital, Beijing, China; 3 School of Medicine, Tsinghua University, Beijing, China

**Keywords:** Gastric cancer, Cancer stem cells

## Abstract

**Background:**

Stearoyl-CoA desaturase-1 (SCD1) is reported to play essential roles in cancer stemness among several cancers. Our previous research revealed significant overexpression of SCD1 in primary gastric cancer stem cells (GCSCs), with its functional role still unknown.

**Methods:**

We stably established three primary GCSCs by sphere-forming assays and flow cytometry. Protein quantification and bioinformatics analysis were performed to reveal the differential protein pattern. Lentivirus-based small-interfering RNA (siRNA) knockdown and pharmacological inhibition approaches were used to characterise the function and molecular mechanism role of SCD1 in the regulation of GC stemness and tumour metastasis capacity both in vitro and in vivo.

**Results:**

SCD1 was found to increase the population of GCSCs, whereas its suppression by an SCD1 inhibitor or knockdown by siRNA attenuated the stemness of GCSCs, including chemotherapy resistance and sphere-forming ability. Furthermore, SCD1 suppression reversed epithelial-to-mesenchymal transition and reduced the GC metastasis probability both in vitro and in vivo. Downregulation of SCD1 in GCSCs was associated with the expression of Yes-associated protein (YAP), a key protein in the Hippo pathway, and nuclear YAP translocation was also blocked by the SCD1 decrease.

**Conclusions:**

SCD1 promotes GCSC stemness through the Hippo/YAP pathway. Targeting SCD1 might be a novel therapeutic strategy, especially to suppress GC metastasis and sensitise chemotherapy.

## Background

Gastric cancer (GC) is the third most commonly diagnosed cancer and the fifth leading cause of cancer mortality worldwide.^1^ Cancer metastasis and therapy failure are considered as the main causes of GC-related deaths,^2^ but the underlying mechanism is poorly understood. Therefore, identifying novel genes/targets and elucidating the molecular mechanisms of progression and metastasis of GC are important.

Recently, mounting evidence has emerged in support of the cancer stem cells (CSCs)/tumour-initiating cell model^3^ for leukaemia^4^ and a wide range of solid tumours,^5–7^ including gastrointestinal cancers.^8–10^ CSCs are now regarded as the GC origin, and are implicated in cancer recurrence and chemotherapy resistance.^11^ Our previous study established stably passaged primary CSCs from GC metastatic sites, which exhibited enhanced stem-like properties and increases in GC stem cell (GCSC) markers.^12^ Considering the fact that dysregulated protein patterns in GCSCs have hardly been investigated,^13^ we performed iTRAQ (isobaric tags for relative quantification) analysis to explore the possible regulation mechanism of GCSCs. As a result, we found that a lipogenesis-associated protein, stearoyl-CoA desaturase-1 (SCD1), was most upregulated. SCD1 is an important enzyme located in the endoplasmic reticulum (ER), which catalyses desaturation of lipids.^14^ However, the role and function of SCD1 in GC or GCSCs have not been clearly elucidated.

In this study, we report the proteomic profiles of intracellular proteins in primary GCSCs and functional analysis of dysregulated proteins. Moreover, we explored the clinical significance of the most upregulated protein, SCD1, and its association with CSC properties. Whether SCD1 promoted stemness was assessed by SCD1 knockdown (SCD1-KD) or SCD1 inhibitor treatment, and its suppression sensitised GC cells to chemotherapy. Furthermore, we explored the mechanism underlying the regulation of SCD1 in GCSCs. Collectively, we investigated the expression pattern of GCSCs and the biological functions of the most upregulated protein, SCD1, providing new insights into the treatment of GC, especially targeting their stem-like properties, such as tumour metastasis and chemotherapy resistance.

## Methods

### Cell culture

The commercial cell lines HGC-27, NCI-87, BGC-7901 and GES-1 were purchased from Shanghai Cell Bank and were cultured in Dulbecco’s modified Eagle’s medium (DMEM, Invitrogen, CA, USA) according to the manufacturer’s instructions. Adherent cells were maintained in a standard condition at 37 °C with 5% CO_2_. Primary GC cells were obtained and cultured as previously described.^12,15^ Briefly, fresh human GC tissues were obtained immediately after resection from patients who underwent gastrectomy in a Chinese PLA General Hospital (PLAGH). All samples were transported to the laboratory on ice within 30 min and disaggregated mechanically, followed by digestion with 1 mg/ml collagenase I and 1 mg/ml collagenase IV (Life Technologies, Waltham, MA, USA) at 37 °C for 1–1.5 h. Tumour digestion was terminated and then seeded into ultra-low-attachment six-well plates with modified DMEM/F12, 2% B27 (Invitrogen), 1% ITS (insulin–transferrin–selenous acid, Corning, NY, USA), epidermal growth factor (20 ng/ml, Peprotech, Hartford, CT, USA), basic fibroblast growth factor (10 ng/ml, Peprotech), leukemia inhibitory factor (10 ng/ml, Peprotech) and gastrin I (10 ng/ml, Peprotech).

This study was approved by the institutional Review Board of the PLAGH, and all patients provided written informed consent. This study was conducted in accordance with the Declaration of Helsinki. The clinicopathological information was extracted from the electronic medical record system.

### Protein extraction and preparation

Cell monolayers (adherent cells) or sphere cells (CSCs) were grown until a confluence of 70–80% in 100-mm plates or ultra-low-attachment six-well plates, and then washed three times with phosphate-buffered saline and harvested with a scraper or by centrifugation. After incubation at 100 °C for 10 min and two consecutive cycles of vortexing, samples were centrifuged at 4 °C for 10 min at 10,000 × *g*. Quantification of protein lysates was measured with a Protein BCA assay Kit (Bio-Rad, CA, USA). Protein extracts from cancer cells were aliquoted and stored at −80 °C until further analysis.

### iTRAQ labelling and relative quantification

Equal amounts of proteins from adherent and sphere cells (200 μg) were precipitated and mixed with 4 μg of trypsin (Sigma-Aldrich, MO, USA) at a final ratio of 1:50, and incubated overnight at room temperature. After protein digestion, peptides were then labelled with iTRAQ reagents into two groups according to the manufacturer’s instructions: one is sphere cells with reagents 126, 127 and 131, and another is adherent cells with reagents 128, 129 and 130. Labelled samples were fractionated using a 75 × 150-μm Zorbax 300SB-C18 column (Microm, Aubrun, CA) in a high-performance liquid chromatography, 20AD HPLC system (Shimadzu, Kyoto, Japan).

A TripleTOF 5600 (Applied Biosystems, CA, USA) coupled with an Easy-nLC1000 system (Thermo Fisher Scientific, USA) was used for protein identification and quantification. Peptides were subjected to nanoelectrospray ionisation tandem mass spectrometry through the TripleTOF 5600 coupled in line to the HPLC system, with an electrospray voltage of 2.2 kV and capillary temperature of 270 °C. The analytical cycle included a MS survey scan and the scan range was 300–1650*m*/*z*.

ProteinPilot 2.0.1 software (Applied Biosystems) and Mascot^TM^ (Matrix Science Inc., Boston, MA, USA) were applied to search against the NCBInr database.

### Database search and bioinformatic analysis

Biological functional analysis of the different modulated proteins detected by iTRAQ quantification was performed according to their functions, biological process and cellular component, using the String 9.0 software.^16^ Differentiated protein expression was considered to be significant when the expression was increased or decreased with a fold change of 1.5 and a *p* value <0.05 in biological replicates (Supplementary Table S1).

### Western blotting

Cells were lysed with RIPA extraction reagent (Beyotime, Beijing, China) supplemented with a protease inhibitor cocktail. Cell protein lysates were separated by sodium dodecyl sulfate–polyacrylamide gel electrophoresis and transferred to nitrocellulose membrane (Millipore) and incubated with specific antibodies, for example, SCD1, Yes-associated protein (YAP) and so on. β-actin antibody was used as the control. Specific brands were detected by enhanced ECL chemiluminescence reagent. Detailed information of antibodies is given in Supplementary Table S2.

### RNA extraction and quantitative real-time RT-PCR

Total RNA was extracted from GC tissues or cancer cells using TRIzol reagent (Invitrogen) according to the manufacturer’s instructions. First-strand complementary DNA (cDNA) was synthesised using HiscriptQ RT SuperMix for quantitative PCR (qPCR) (Vazyme, Nanjing, China) on a 7900HT system (Applied Biosystem, Waltham, MA, USA). The PCR primers used to amplify target genes are shown in Supplementary Table S3. The results were normalised to the expression of β-actin, and the relative levels of messenger RNA (mRNA) were analysed by the 2^−△△CT^ method; each sample was analysed in triplicate.

### Immunohistochemical staining

Immunohistochemical staining was performed on 93 cases of GC and their paired adjacent tissues according to the standard method described previously using the following antibodies: anti-SCD1 (ab19862, 1:100, Abcam, Cambridge, UK), anti-YAP1 (14074S, 1:200, Cell Signaling Technology (CST), Danvers, MA, USA), anti-E-cadherin (14472S, 1:100, CST), anti-vimentin (5741S, 1:20, CST), anti-N-cadherin (13116S, 1:100, CST) and anti-TEA domain transcription factor 1 (TEAD1) (ab133533, 1:100, Abcam). Immunohistochemical (IHC) staining scores were evaluated based on the ratio and intensity of stained cells following the methods described in the previous study.^12^ A categorisation of expression into three groups was based on the scores: negative, moderate and positive expression

### Spheroid-forming assay

Digested cells from GCs or GC cell lines were cultured to form spheres in ultra-low-attachment six-well plates (Corning) at a density of 10,000 cells/ml with modified medium described above. All cells were routinely checked for mycoplasma contamination with PCR test. The primary cells were cultured for at least 3 weeks or until the appearance of tumourspheres (diameter >100 μm).

### Annexin V apoptosis and cell-cycle assays

Cell apoptosis was measured 48 h after treating with A939572 or infected with lentivirus using the APC-Annexin V Apoptosis Detection Kit (BD Bioscience, NJ, USA) according to the manufacturer’s instruction. After double staining with APC-Annexin V and propidium iodide (PI), the cells were analysed with a flow cytometer (FACScan, BD Bioscience). The apoptosis results were then extracted and analysed by the FlowJo 10.0.7 software (FlowJo, Ashland, OR, USA).

Cells for cell-cycle analysis were stained with PI using DNA labelling solution kit (Cytognos, Spain) following the protocol, and were analysed by FACScan. The percentages of the cells in G0–G1, S and G2–M phase were counted and compared using Modfit LT 3.1 (Verity Software, Topsham, ME, USA.)

### Gene-set enrichment assays

We downloaded The Cancer Genome Atlas (TCGA) Stomach Adenocarcinoma HTSeq-FPKM data using UCSC Xena (http://xena.ucsc.edu). A total of 370 GC cancerous tissues had RNA sequence data. GSEA was performed to compare the differences in molecular pathways in cell process between the low and high SCD1 expression groups (cut-off at median value) on the data from stomach adenocarcinoma dataset of TCGA, following the protocols of the description by Subramanian et al.^17^ GSEA was conducted using GSEA 3.0.0 (http://www.broadinstitute.org/gsea/).

### Lentivirus construction and transfection

For lentivirus construction, oligonucleotides with targeting sequences were used for the cloning of small-interfering RNA (siRNA) in the hU6-MCS-CMV-puro lentiviral vectors (GeneChem Co., Shanghai, China). The three recombinant lentiviruses with siRNA-SCD1 were produced by co-transfection of 293T cells with plasmids pHelper 1.0 and pHelper 2.0 (GeneChem Co.). Lentivirus-containing supernatant was harvested 48 h after transfection and concentrated by ultracentrifugation (2 h at 50,000 × *g*). The detailed sequences of target siRNA are listed in Supplementary Table S4. Transfection of the siRNAs was performed with Hitrans G (GeneChem) according to the manufacturer’s instruction. Stably transfecting clones were validated by quantitative real-time RT-PCR (qRT-PCR).

Overexpression plasmids and siRNAs of YAP were transfected into cells using Lipofectamine 2000 (Invitrogen). The knockdown sequence of YAP was as follows: siYAP, 5′-GACATCTTCTGGTCAGAGA-3′. A full-length YAP cDNA was synthesised by GeneChem Company (Shanghai, China), and cloned into plasmid-CMV/MCS//SV40/Neomycin. At 48 h post transfection, the modified cells were harvested for western blot and qRT-PCR validation.

### Cell proliferation and chemotherapy sensitivity analysis

Cell proliferation assay was performed with Cell Counting Kit-8 (CCK-8, Dojindo, Kumamoto, Japan). The cells were grown in 96-well plates and cultured at 37 °C and 5% CO_2_ atmosphere. The cells were plated in DMEM-F12 (Invitrogen) at a density of 5000 cells per well in 96-well plates. CCK-8 assays were performed at 0, 24, 48 and 72 h after being seeding. CCK-8 reagent (10 μl) was added to each well, and the cells were incubated for 1 h at 37 °C.

The modified cells were treated with chemotherapy reagents, including 25 μg/ml 5-fluorouracil (5-Fu) and 10 μg/ml oxaliplatin (Sigma-Aldrich, St. Louis, MO, USA). Viable cell counts were estimated by CCK-8 assays by measuring the optical density at 450 μm.

### In vivo tumorigenicity and tumour metastasis assay

Six-week-old male severe combined immunodeficiency (SCID) mice were purchased from Vital River Laboratory Animal Technology (Beijing, China) and raised in an accredited Specific Pathogen Free Animal Facility at Chinese PLA General Hospital. All protocols were approved by the Institutional Animal Care and Use Committee of the Chinese PLA General Hospital.

Twenty SCID mice were randomly divided into the following four groups (five mice per group), which included suspensions of modified HSC034 cells: (1) negative control siRNA (siNC), (2) SCD1-KD, (3) siNC + A939572 (5 μM/ml) and (4) SCD1-KD + oxaliplatin (10 μg/ml) cells (1 × 10^6^ cells) by injecting into the rear flank of mice. Tumour growth was evaluated using a Vernier calliper every 2 days, and the tumour volume was calculated with the formula: *V* = 0.5 × length × width^2^.

The aforementioned modified cancer cells were also injected through tail veins of 20 mice (randomly divided into four equal-sized groups). Mice were inspected every 2 days and sacrificed by isoflurane anaesthesia and cervical dislocation at 4 months after injection. Solid tumours and organs were removed and examined in vision and under a microscope. Tumours were then fixed by formalin for 24 h and then embedded in paraffin.

### Statistical analysis

The results obtained from qPCR, spheroid formation assays, flow cytometry analysis, invasion and migration assays were determined by two-sided Student’s *t* test. Numeric variables were shown as the means with their standard deviations. Pearson’s *χ*^2^ test was applied to assess the association between SCD1 status and clinicopathological variables. Pearson’s correlation analysis was used to calculate the correlation between SCD1 and YAP, TEAD1. All statistical analyses were performed using the SPSS 25.0 software (SPSS Inc., Chicago, IL, USA). Survival curves of progression-free survival (PFS) and overall survival (OS) were drawn using the Kaplan–Meier method, and the statistical significance was calculated by log-rank test. A *p* value <0.05 determined the statistical significance.

## Results

### Differential expression profiles of the whole proteome in GCSCs revealed by protein quantification and bioinformatic analysis

**Fig. 1:**
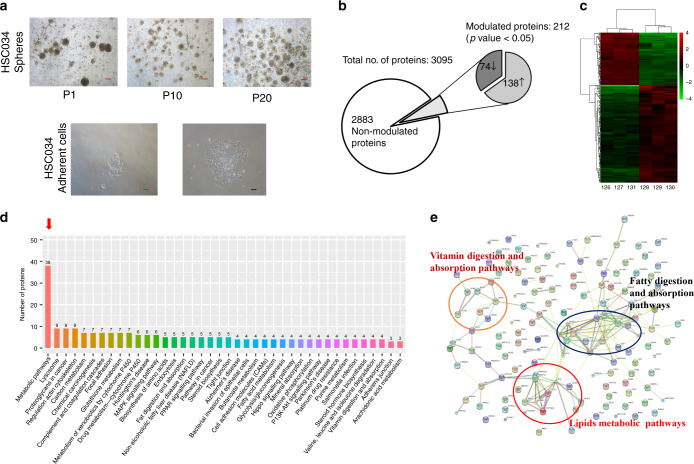
Isobaric tags of relative quantification (iTRAQ) and bioinformatic analysis of modulated proteins in gastric cancer sphere cells (GCSCs) compared with differentiated cells. **a** Representative images showing different morphology of sphere and differentiated GC cells (scale bar, 10 μm in black and 25 μm in red; P, cell passage). **b** Summary of identified protein by iTRAQ between GCSCs and differentiated cancer cells. **c** Heatmap of a modulated cluster of GCSCs (left panels) compared with differentiated cancer cells (right panels). **d** Top 40 enrichment pathways according to KEGG pathway analysis (arrow indicated the most enriched pathway). **e** Protein interactions of upregulated GCSCs according to the STRING database.

In our previous study, we established GC stem-like cells (GCSLCs) from GC hepatic metastasis sites by sphere-forming assays.^12^ After serial passaging (Fig. 1a), as well as flow cytometric analyses, we determined their stemness as the population of GCSCs. To explore GC stemness-associated protein profiles, we performed iTRAQ-based proteomics analysis of three GCSC lines and their differentiated cells (Fig. 1b, c). As a result, 3095 proteins or peptides were identified, including 2883 non-significantly modulated proteins and 212 dysregulated proteins (Supplementary Table S1). Among them, 74 were downregulated and 138 were upregulated.

The list of modulated proteins was used to investigate the molecular pathways altered in GCSCs by String 9.0 analysis. As a result, the most striking pathways involving the upregulated proteins in GCSCs compared with differentiated cells were lipid metabolic pathways (Fig. 1d). Correspondingly, protein network analysis also showed an important role of lipid metabolic pathways in the dysregulated protein interactions (Fig. 1e).

### SCD1 is upregulated in metastatic GCs/GCSCs and associated with poor prognoses

**Fig. 2:**
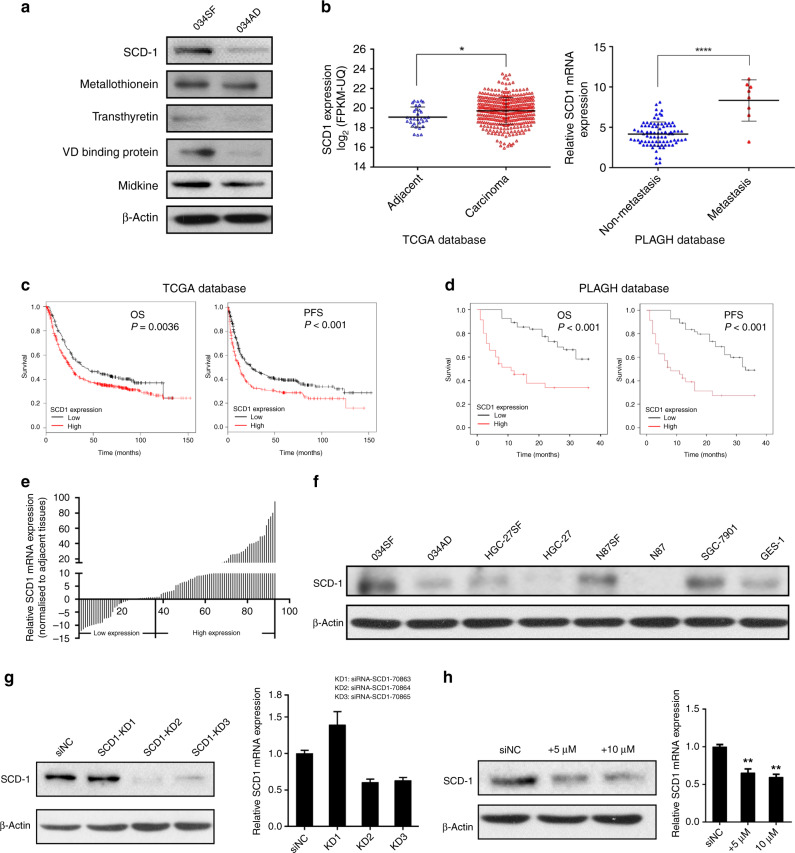
SCD1 expression is upregulated in gastric cancer and associated with poor prognosis. **a** Validation of top five proteins identified by iTRAQ analysis with western blotting. **b** Relative expression of SCD1 in metastatic cancer sites, gastric cancer tissues and adjacent non-tumour tissues from the TCGA and PLAGH databases. **c**, **d** Overexpression of SCD1 protein predicts worse survival of GC patients both in the TCGA and PLAGH databases (statistical significance was evaluated by log-rank test, *p < 0.05, ****p < 0.0001). **e** SCD1 expression was analysed by qRT-PCR in gastric cancer tissue and the corresponding adjacent tissues, and the data were displayed as the △Ct value (*n* = 93). **f** Western blotting of SCD1 protein in gastric cancer cell lines and the normal gastric epithelium cell line (GES-1). **g** WB and qRT-PCR analysis of SCD1 expression in SCD1–KD1, SCD1–KD2 and SCD1–KD3 HSC034 cells and in control. **h** WB and qRT-PCR analysis of SCD1 expression in HSC034 cells treated with A939572 (0, 5 and 10 μM/ml). Error bars indicate standard deviation (SD) of the mean value. *p < 0.05 and **p < 0.01.

Among the overexpressed proteins, SCD1 was found to be the most upregulated (Supplementary Table S1, fold change = 7.23, *p* < 0.001). SCD1 is an important enzyme located in the ER, which catalyses desaturation of lipids and thus participates in the lipid metabolic process.^14^ To verify the expression of dysregulated proteins in GCSCs, we performed western blotting and qRT-PCR analysis of the most five upregulated proteins in GCSCs and their corresponding differentiated cells. The results showed that the protein levels of these molecules were in accordance with the results of the iTRAQ analysis (Fig. 2a). Immunohistochemical staining was also performed to examine the protein expression level of SCD1 in patient samples. The results showed moderate-to-strong expression of SCD1 in the cytoplasm and nucleus (Supplementary Fig. S1a). Combining the validation results with the bioinformatic analysis, we therefore chose the most upregulated protein (SCD1) as the main target protein in this study.

To identify the role of SCD1 in GC and GCSCs, we analysed the mRNA sequencing data of 376 GC tissues (GC group) and 37 adjacent gastric mucous tissues (adjacent group) in TCGA. We found that SCD1 transcriptional levels were higher in tumour tissues than in adjacent non-tumour tissues (*p* = 0.01, Fig. 2b, left panel). We also examined SCD1 mRNA expression in samples of primary cancers and metastatic sites from our institute by qRT-PCR. The results showed more significantly increased expression in metastatic GC tissues than in primary tumours (*p* < 0.001, Fig. 2b, right panel).

The associations of SCD1 mRNA expression and the major clinicopathological features of the 93 GC cases are presented in Supplementary Table S5. Overexpression of SCD1 compared with adjacent normal tissues was significantly associated with the pTNM stage (*p* < 0.001) and distant metastasis (*p* = 0.038). In terms of prognosis prediction, upregulation of SCD1 was also a negative predictor for both OS and PFS in TCGA and PLAGH databases (Fig. 2c, d).

We then examined 93 GC patient specimens and analysed the expression of SCD1 in tumour tissues and their adjacent counterparts by qPCR analysis (Fig. 2e). Approximately 58% of patients showed upregulation of SCD1 mRNA in their tumour tissues compared with non-tumour counterparts.

To further examine whether SCD1 downregulation functionally regulated the traits of GCSCs, we performed both SCD1-KD experiment using a lentivirus-based approach and pharmacological inhibition. First, western blot analysis showed relatively high expression of SCD1 in primary GC cell line HSC034SF (034SF) among a panel of GC cell lines and a gastric mucosal cell line (GES-1) (Fig. 2f). Therefore, the HSC034 cell line was chosen for KD and further experiments. The HSC034 cell line was transfected with siNC and three SCD1-KD siRNAs. Expression levels of SCD1 were assessed by western blot analysis and qPCR (Fig. 2g). The results showed significant decreases of SCD1 protein in SCD1–KD2 and SCD1–KD3 groups, between which the SCD1–KD2 group had the highest decrease. Therefore, the SCD1–KD2 group was chosen for further experiments. Moreover, we used A939572 (MCE) as the pharmacological inhibitor to suppress SCD1 expression.^18^ Both concentrations of 5 and 10 μM/ml MCE inhibited SCD1 expression efficiently (Fig. 2h).

### SCD1 regulates self-renewal, chemoresistance and stem cell marker expression of GC cells

**Fig. 3:**
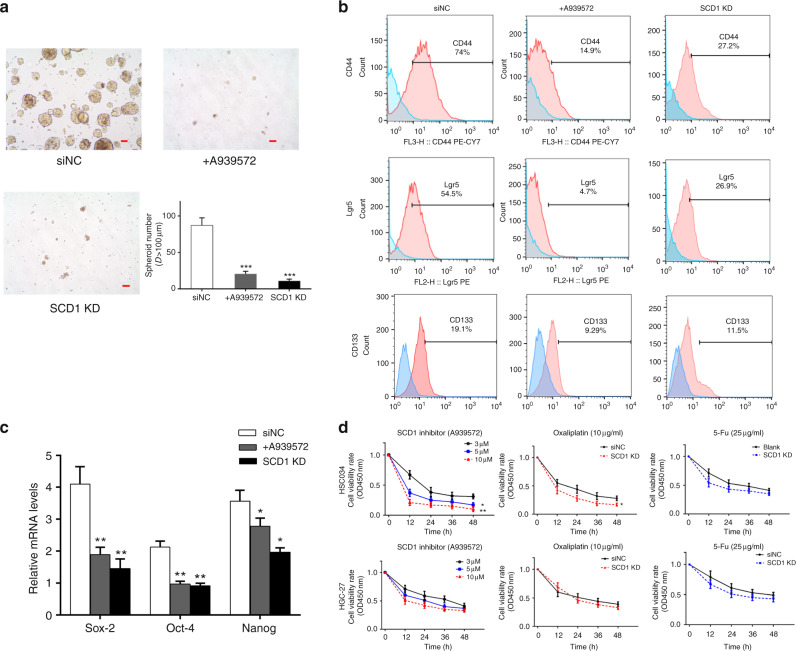
Downregulation of SCD1 attenuated the stem-like properties of gastric cancer stem cells. **a** Knockdown or inhibition of SCD1 reduced the size and number of sphere cells formed by HSC034 cells (scale bar, 25 μm in red). **b** Knockdown or inhibition of SCD1 decreased cell surface marker expression of CD44, Lgr5 and CD133 in HSC034 cells. **c** qRT-PCR showed decreased trends in transcriptional levels of stemness markers, Sox-2, Oct-4 and Nanog. **d** CCK-8 assays showed that the cell viability was suppressed by SCD1 inhibition and oxaliplatin in HSC034 cells but not in HGC-27. Values represent the mean ± SD of three independent experiments. *p < 0.05, **p < 0.01 and ***p<0.001, respectively, *t* test.

To determine whether SCD1 regulated the self-renewal of GCSCs, control cells and SCD1-KD- or A939572-treated cells were subjected to a spheroid formation assay. Compared with control cells, significantly less and smaller spheroids were observed in assays of treated cells (Fig. 3a). To further investigate whether SCD1 regulates the stem cell population in GC cells, we measured and compared the expression of three stem cell markers, CD44,^9^ Lgr5^19,20^ and CD133,^21^ in SCD1-KD cells and their controls by flow cytometry. Transcriptional stemness markers, such as Sox-2, Oct-4 and Nanog, were also measured by qRT-PCR.^22^ We found that SCD1-KD- and A939572-treated cells exhibited significant decreases in those markers (Fig. 3b, c).

Another important biological feature of CSCs is resistance to chemotherapy.^23^ We therefore compared the viability of two cell lines treated with commonly used chemotherapeutic drugs for GC, oxaliplatin and 5-Fu, by CCK-8 assays. HSC034 cells showed lower viability as the concentration of the A939572 was increased. However, in another cell line with low SCD1 expression, HGC-27, A939572 did not significantly affect cell proliferation (Fig. 3d). HSC034 cells were more sensitive to oxaliplatin when SCD1 was downregulated, whereas no significant changes were detected in HGC-27- or 5-Fu-treated cells.

### SCD1 regulates invasion, migration and epithelial-to-mesenchymal transition of GC cells

**Fig. 4:**
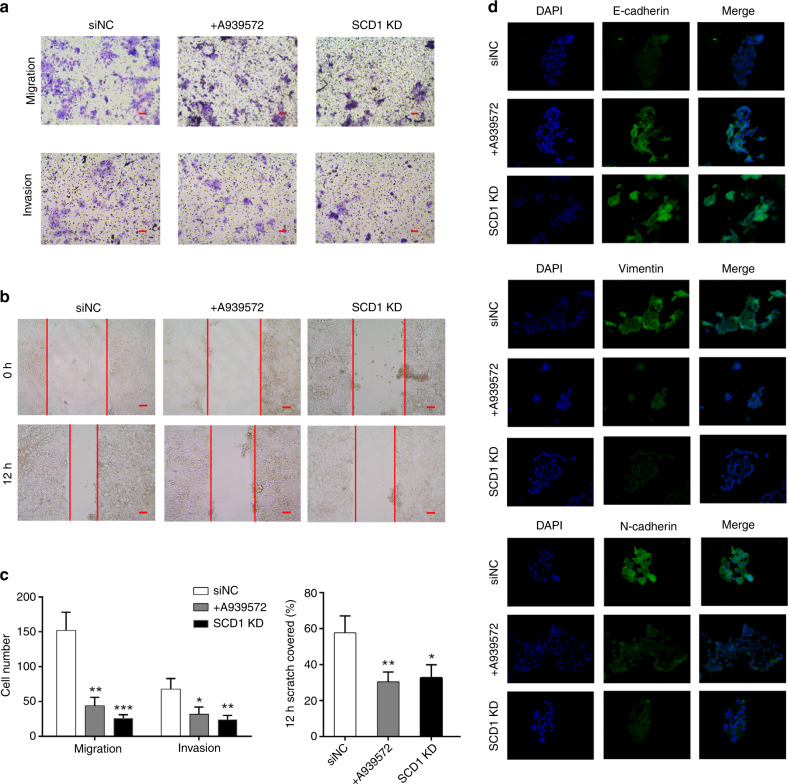
Effect of SCD1 downregulation on gastric cancer cell invasion, migration and EMT phenotype. **a** Transwell migration and invasion assays demonstrated the decreased migratory and invasive abilities of SCD1-KD cells and cells treated with A939572. **b** Wound-healing ability was attenuated by SCD1 downregulation. **c** Quantification of transwell and would-healing assays. (Values represent the mean ± SD of three independent experiments. *p < 0.05, **p < 0.01  and ***p < 0.001, respectively, *t* test.) **d** Immunofluorescence assays showed that downregulation of SCD1 led to an increase of E-cadherin but a decrease in vimentin and N-cadherin expression (scale bar, 10 μm in black and 25 μm in red).

A major feature of CSCs is their high metastatic potential. Matrigel-coated invasion and Matrigel-uncoated migration transwell assays showed that SCD1 downregulation in HSC034 cells impaired their invasiveness and migratory potential (Fig. 4a). Downregulation of SCD1 also suppressed the wound-healing ability of HSC034 cells compared with control cells (Fig. 4b, c). IHC staining in the GC tissues demonstrated that overexpression of SCD1 was associated with the expression of mesenchymal markers, vimentin and N-cadherin, but negatively correlated with the expression of E-cadherin (Supplementary Fig. S1c). Furthermore, knockdown of SCD1 in HSC034 cells reversed the molecular changes in E-cadherin, vimentin and N-cadherin expression, which revealed a more epithelial cell-like phenotype (Fig. 4d).

### SCD1 downregulation impairs YAP expression and nuclear translocation, and leads to G1 arrest of GC cells

**Fig. 5:**
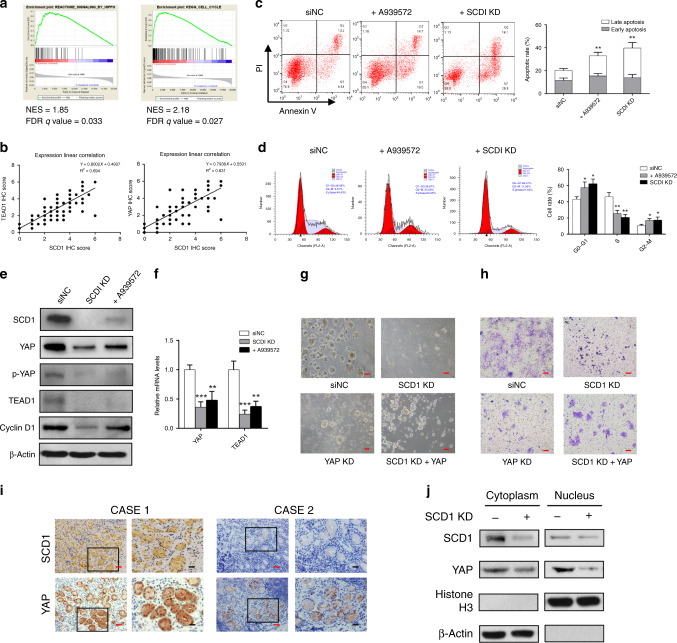
SCD1 affected cell stemness via Hippo–YAP pathway and cell-cycle-associated pathway. **a** GSEA analysis indicated significantly enhanced expression of Hippo and cell-cycle pathway genes in gastric cancer from the TCGA database. **b** YAP and TEAD1 expressions were positively correlated with SCD1 protein expression (statistical significance was evaluated by Pearson’s correlation test). **c** Suppression of SCD1 expression increased the apoptosis rates of HSC034 cells. **d** The SCD1 downregulation by siRNA knockdown or A939572 led to a significant increase in the G0–G1 phase and a decrease in the S phase, respectively. **e** Western blotting assays showed a significant decrease in the Hippo/YAP pathway key proteins, YAP, p-YAP and TEAD1, and the cell-cycle-dependent protein, cyclin D1 after SCD1 downregulation. **f** qRT-PCR assay showed transcriptional decline in YAP and TEAD1 mRNA after SCD1 downregulation. **g**, **h** The sphere-forming and cell invasion ability of HSC034 cells were impaired by SCD1 or YAP knockdown, which could be partially reversed by YAP overexpression (scale bar, 25 μm in red). **i** Representative images of immunohistochemical staining (IHC) showed weakened nucleus YAP expression when SCD1 is negatively expressed (scale bar, 10 μm in black and 25 μm in red). **j** Nuclear YAP expression was more significantly decreased, whereas cytoplasmic YAP levels were slightly reduced after SCD1 knockdown. Error bars indicate standard deviation (SD) of the mean value. *p < 0.05, **p < 0.01 and ***p<0.001, respectively, *t* test.

SCD1 was recently reported to be involved in the activation of several oncogenic signalling pathways (e.g. the Wnt/β-catenin pathway) in other cancers.^24^ Therefore, to explore the targeted pathways involved in SCD1 regulating stemness of gastric cancer, we performed GSEA of common tumour-related pathways. The GSEA results showed distinct upregulation of the Hippo/YAP pathway (Fig. 5a, normalised enrichment score (NES) = 1.85, false discovery rate (FDR) *q* value = 0.033) and cell cycle pathway (NES = 2.18, FDR *q* value=0.027). Therefore, we explored the correlation between SCD1 and Hippo pathway. IHC scores showed that in GC tissues, SCD1 was correlated with the expression of two key Hippo pathway proteins, TEAD1 and YAP (Fig. 5b).^25^ In addition, cell cycle and apoptosis analyses were performed to explore the function of SCD1. As a result, we found that HSC034 cells treated with A939572 and SCD1-KD cells had higher apoptotic rates than siNC cells (Fig. 5c). Moreover, cells treated with A939572 and SCD1-KD cells showed higher cell-cycle arrest with significantly more cells in the G1/G0 phase and fewer cells in the G2/S phase of the cell cycle compared with siNC cells (Fig. 5d). Western blot analysis also showed that the level of the key G1/S-transition protein cyclin D1 was decreased accordingly (Fig. 5e). The protein levels of YAP, phosphorylated YAP (p-YAP) and TEAD1 were also decreased by SCD1-KD or treatment with A939572 (Fig. 5e). qRT-PCR assay revealed that the transcriptional level of YAP and TEAD1 mRNA was decreased after SCD1-KD (Fig. 5f).

To further determine the role of YAP in SCD1 regulating the stemness of GCSCs, YAP–siRNA was transfected into HSC034 cells to suppress the YAP expression. Subsequent sphere-forming and transwell assays found that YAP knockdown could lead to similar inhibitory effect on self-renewal and invasion capacity as SCD1-KD did. On the other hand, overexpression of YAP in HSC034 cells tends to partially reverse the inhibitory effect that was induced by SCD1-KD (Fig. 5g, h).

Moreover, IHC staining showed that nuclear expression of YAP was decreased when SCD1 was almost absent (Fig. 5i). We next simultaneously examined nuclear and cytoplasmic expression of YAP in HSC034 cells when SCD1 was downregulated, and found that the protein level of YAP was significantly decreased in the nucleus, but not in the cytoplasm (Fig. 5j).

### SCD1 downregulation attenuates GCSC tumorigenicity and the metastasis-dependent Hippo–YAP pathway in vivo

**Fig. 6:**
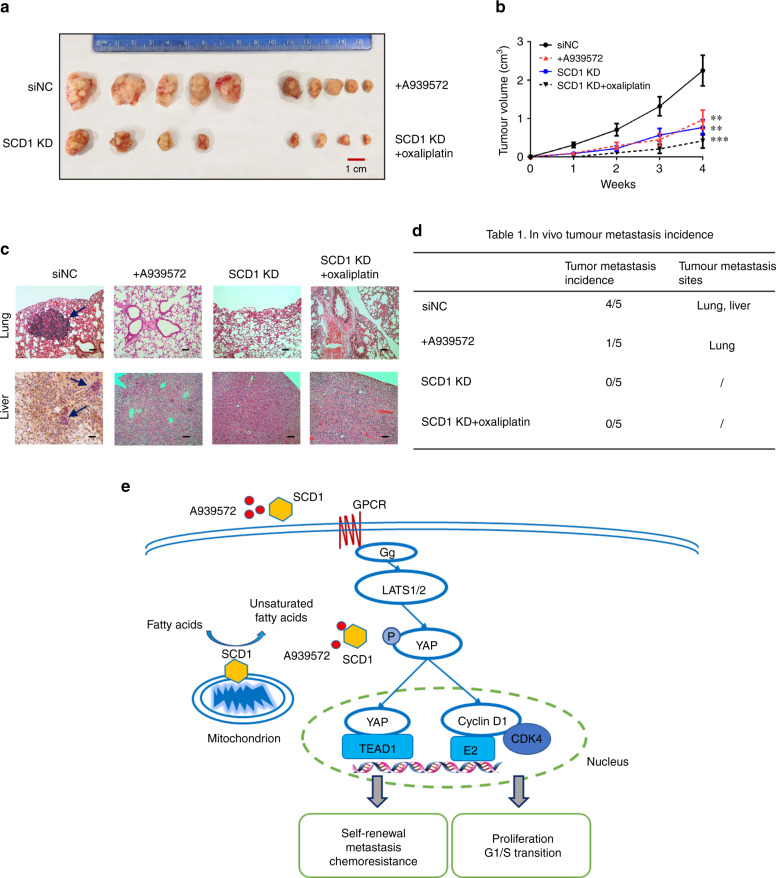
Effect of SCD1 inhibition on gastric cancer cell tumorigenicity and metastasis in vivo. **a** In vivo assay of the effect of SCD1 suppression and chemotherapy on gastric cancer cell tumorigenicity. **b** Statistical analysis of tumour growth volume of xenografts formed by modified HSC034 cells in 4 weeks (values represent the mean ± SD of three independent experiments. **p < 0.01 and ***p < 0.001). **c** Representative images of haematoxylin and eosin (HE) staining of mice lung and liver tissues following different cell treatments (scale bar, 10 μm in black and 25 μm in red; arrow, metastatic sites). **d** The ratios of lung and liver metastatic sites detected after tail-vein injection by control or modified HSC034 cells. **e** Proposed schematic mechanism of SCD1 regulating stem-like properties in gastric cancer stem cells via Hippo/YAP pathway and the effect of SCD1-targeted inhibition.

To determine whether SCD1 affects tumour growth and metastasis in vivo, SCD1-KD HSC034 cells and cells treated with A939572 or oxaliplatin were injected subcutaneously or via the tail vein into SCID mice. All mice developed tumours at the injection sites afterwards, except two did not (one in the SCD1-KD and one in the SCD1-KD each treated with oxaliplatin group). As a result, the average size of tumours generated by SCD1-KD cells and cells treated with the SCD1 inhibitor was significantly smaller than those generated by control cells (Fig. 6a, b). The tumour metastasis models established by tail-vein injection had higher rates of liver and lung metastases when injected with untreated HSC034 cells, whereas SCD1-KD cells and cells treated with the SCD1 inhibitor/chemotherapy led to lower development of metastatic tumour sites (Fig. 6c, d).

## Discussion

Recently, mounting evidence has suggested that CSCs play an essential role in cancer initiation and progression.^11,26^ Proteomics has been adopted as a powerful method to efficiently determine protein networks responsible for CSC pathophysiology and elucidate the molecular mechanisms of CSCs.^27^ In the present study, we employed proteomics analysis to explore the key functional proteins in GCSCs and the involved pathways, as well as the underlying mechanism. Interestingly, metabolic pathways, especially lipogenesis pathways, were highly upregulated in GCSCs compared with differentiated cells. Lipogenesis is vital to maintain the stemness properties of cancers.^28^ When we examined the dysregulated proteins in detail, expression of SCD1 was ranked as the highest. SCD1 has been widely studied in metabolic diseases, such as diabetes and obesity.^29,30^ In addition, some studies have indicated the biological role of SCD1 in solid tumours, such as hepatocellular cancer and lung cancer,^31,32^ either through ER stress or the Hippo/Yap pathway. Although evidence suggests that SCD1 is important for cancer progression and stemness properties, the role of SCD1 in GCSCs remains to be investigated, especially in terms of metastasis and chemotherapy failure.

qPCR and western blot analysis showed that SCD1 was highly expressed in GCSCs and more than 58% of GC patients. In SLCs derived from patients and tumour tissues of metastatic cases, SCD1 expression was more predominant. Furthermore, upregulated SCD1 protein expression in GC patients was significantly correlated with their worse survival. Downregulation approaches for SCD1 in vitro and in vivo were then used to examine the functional role of SCD1 in GCSCs. First, SCD1 was found to play a regulatory role in GCSCs, including regulating expression of surface markers CD44, CD133 and Lgr5, and the sphere-forming ability of GCSCs. Second, we found that SCD1 inhibition or knockdown increased some chemotherapeutic effects in GC cells with high SCD1 expression. In addition, SCD1 suppression attenuated the migration/invasion and wound-healing ability of GC cells. Consistently, overexpression of SCD1 was correlated with the presence of mesenchymal markers in GC tissues, while GC cells demonstrated mesenchymal-to-epithelial transition phenotype after SCD1 downregulation. Notably, the in vivo xenograft assays also confirmed the inhibitory effects on tumour growth and metastasis after SCD1 downregulation.

Next, we elucidated the downstream mechanism of SCD1 by several bioinformatics analyses. Although a relationship between SCD1 and the Hippo pathway has been found in other CSCs,^32,33^ the precise role of SCD1 in GC has not yet been addressed. Our study revealed the protein relevance of SCD1 and the Hippo pathway, as well as a cell-cycle-related pathway. Subsequent experiments demonstrated that SCD1 suppression also led to G1 arrest and a higher apoptosis rate in GC cells. We showed for the first time in this study that key proteins of the Hippo signalling pathway, YAP and TEAD1, were correlated with SCD1 expression, and SCD1 inhibition led to YAP suppression. In addition, SCD1 downregulation decreased the expression of p-YAP and caused YAP disassembly in GC cell nuclei, which also attenuated the Hippo pathway activation. Further rescue assays revealed that overexpression of YAP could partially reverse the inhibitory effect on GCSCs induced by SCD1-KD. Taken together, these results suggested that SCD1 promotes the GC stemness through the Hippo/YAP pathway.

The highlight of this study lies in uncovering the functional role of SCD1 in inhibiting the tumour invasion/metastasis potential and chemotherapy resistance both in vivo and in vitro. Furthermore, we found that A939572 suppressed self-renewal, migration/invasion and chemotherapy resistance of GC cells, which makes SCD1 an attractive therapeutic target. Inhibiting genes controlling cell lipogenesis, such as SCD1, has been reported to overcome therapeutic resistance in various cancers.^34,35^ Interestingly, in this study, we found that oxaliplatin rather than 5-Fu sensitised SCD1-KD cells to chemotherapy. This may indicate that oxaliplatin is a cell-cycle-independent drug that augments the efficacy of cell-cycle arrest caused by SCD1 downregulation. Therefore, oxaliplatin combined with A939572 in SCD1-overexpressing GC patients might achieve better inhibition of tumour growth and metastasis.

In conclusion, we identified a novel GCSC-associated protein, SCD1, and found that SCD1 regulates stemness, including tumorigenesis, chemoresistance and metastasis, via Hippo/YAP pathways in this study (Fig. 6e). Targeting GCSCs with an SCD1 inhibitor in combination with cell-cycle-independent chemotherapy appears to be a promising, novel therapeutic strategy for treating GC, especially to prevent tumour metastasis and sensitise to chemotherapy.

## Supplementary information

Supplementary materials

## Data Availability

All data generated or analysed during this study are included either in this article or in the supplementary information.
