# Supplementary material for: Stearoyl-CoA-desaturase-1 regulates gastric cancer stem-like properties and promotes tumour metastasis via Hippo/YAP pathway
Source: Br J Cancer. 2020 Apr 30;122(12):1837–47. doi: 10.1038/s41416-020-0827-5 (PMC7283337; doi:10.1038/s41416-020-0827-5)
Supplement: Supplementary file 1 — Supplementary materials [file 41416_2020_827_MOESM1_ESM.pdf]

Supplementary information

Figure S1. Representative IHC staining images included in the study. **a** SCD1 expression in gastric cancer tissues. **b** SCD1 expression in xenograft tissues from siNC and SCD1 KD group. **c** The expression of SCD1 and EMT markers, E-cadherin, Vimentin and N-cadherin in gastric cancer tissues. (Scale bar, 10μm in black and 25μm in red).

Table S1. All modulated proteins ($p<0.05$) in GCSCs comparing to differentiated GC cells.

Table S2. Detailed information of antibodies in western blotting.

Table S3. Primer sequences applied in qRT-PCR.

Table S4. Primer sequences applied in the construction of lentivirus-siRNA.

Table S5. Clinicopathological variables and expression of SCD1 in PLAGH gastric cancer database.

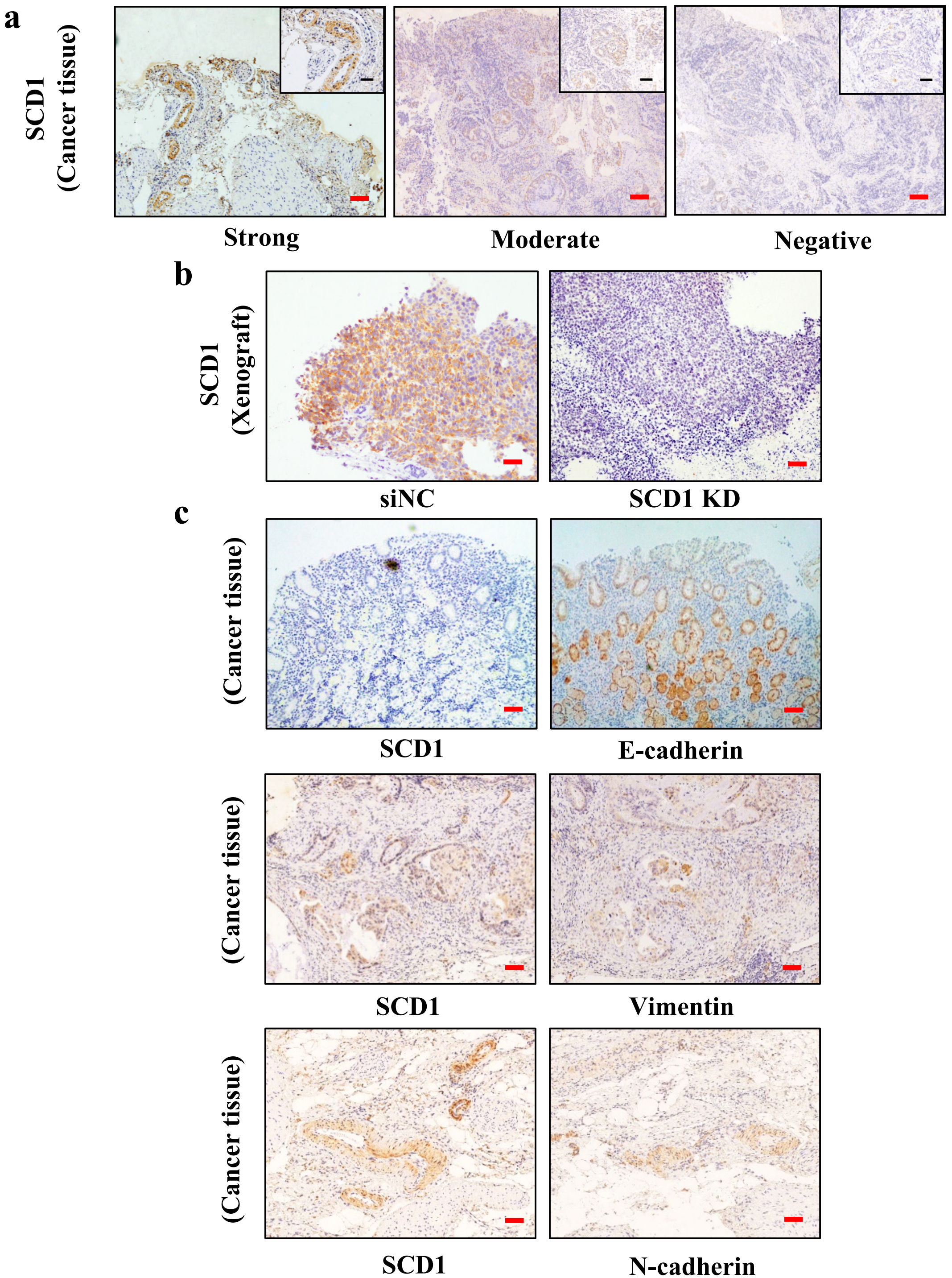
a
SCD1
(Cancer tissue)
Strong
Moderate
Negative
b
SCD1
(Xenograft)
siNC
SCD1 KD
c
(Cancer tissue)
SCD1
E-cadherin
(Cancer tissue)
SCD1
Vimentin
(Cancer tissue)
SCD1
N-cadherin

Table S1. All modulated proteins (p<0.05) in GCSCs comparing to differentiated GC cells.

Number	Protein names	pvalue	Foldchange	regulated
1	Stearoyl-CoA desaturase variant (Fragment)	0.000773	7.230452675	up
2	Metallothionein	0.039018	5.675194661	up
3	Transthyretin	2.56E-05	3.325883201	up
4	Vitamin D-binding protein	0.006704	3.100478469	up
5	Midkine	0.00166	2.939592909	up
6	Apolipoprotein A-I, isoform CRA_a	0.010272	2.846153846	up
7	Retinol-binding protein 2 (Cellular retinol-binding	0.048244	2.775330396	up
8	Pyruvate kinase (EC 2.7.1.40) (Fragment)	0.01614	2.70203578	up
9	cDNA FLJ77911, highly similar to Homo sapiens glu	0.02041	2.685503686	up
10	cDNA, FLJ93585, highly similar to Homo sapiens P	0.002526	2.50262697	up
11	Epididymis secretory sperm binding protein Li 44a	0.000362	2.436998855	up
12	Lysozyme C (EC 3.2.1.17) (1,4-beta-N-acetylmuran	0.010585	2.413538111	up
13	Hydroxymethylglutaryl-CoA synthase, cytoplasmic	0.000491	2.27744402	up
14	Ornithine carbamoyltransferase, mitochondrial (E	6.95E-06	2.274563319	up
15	Lipase	0.000897	2.21888412	up
16	X-prolyl aminopeptidase (Aminopeptidase P) 2, m	2.13E-05	2.209202782	up
17	NADH dehydrogenase [ubiquinone] 1 alpha subco	0.030582	2.188097768	up
18	Protein S isoform 2 (Fragment)	0.011494	2.160695469	up
19	Cytochrome c oxidase assembly factor 3 homolog	3.43E-05	2.150656168	up
20	Mitochondrial ribosomal protein L44, isoform CRA	0.040647	2.140763998	up
21	Epididymis secretory protein Li 4	0.01778	2.10880829	up
22	Lanosterol 14-alpha demethylase	3.88E-05	2.105590062	up
23	Angiotensinogen variant (Fragment)	0.009687	2.100258398	up
24	Ribonuclease T2	0.000141	2.094378546	up
25	Hydroxymethylglutaryl-CoA synthase, mitochondr	0.000872	2.063840654	up
26	Methylsterol monooxygenase 1 (EC 1.14.13.72) (C	0.008659	2.034901366	up
27	Valacyclovir hydrolase (VACVase) (Valacyclovirase	0.001474	2.026222895	up
28	Hydroxyacyl-coenzyme A dehydrogenase, mitocho	0.003075	2.017094017	up
29	cDNA FLJ75087, highly similar to Homo sapiens sa	0.00025	1.98111332	up
30	Espin (Autosomal recessive deafness type 36 prot	0.000739	1.96785361	up
31	Clusterin (Aging-associated gene 4 protein) (Apoli	0.000376	1.960532807	up
32	Metallothionein-1E (MT-1E) (Metallothionein-IE) (	0.027575	1.960039467	up
33	Epididymis secretory sperm binding protein Li 71p	0.000292	1.954702117	up
34	Folate receptor 1 (Adult), isoform CRA_a	0.028891	1.918774319	up
35	Dipeptidyl peptidase 1 (EC 3.4.14.1) (Cathepsin C)	0.004503	1.908385846	up
36	cDNA FLJ39160 fis, clone OCBBF2002290, highly si	0.014824	1.901353965	up
37	PRP4 pre-mRNA processing factor 4 homolog B (Y	9.06E-05	1.900870406	up
38	3'-phosphoadenosine 5'-phosphosulfate synthase	0.000142	1.897151135	up
39	Probable ATP-dependent RNA helicase DDX52 (EC	0.000852	1.895320791	up
40	Methyltransferase-like protein 7B	0.002134	1.885096154	up
41	Sulfotransferase (EC 2.8.2.-) (Fragment)	0.003111	1.866698519	up
42	Visinin-like protein 1 (VILIP) (VLP-1) (Hippocalcin-l	5.24E-05	1.865329513	up
43	Peptidyl-prolyl cis-trans isomerase G (PPIase G) (P	0.008718	1.863072519	up
44	Tetraspanin-3 (Tspan-3) (Tetraspanin TM4-A) (Tra	0.014777	1.857142857	up
45	HCG1773630 (cDNA, FLJ92308, Homo sapiens adi	0.001634	1.856258924	up
46	Fibronectin 1, isoform CRA_n	0.017926	1.848125297	up
47	Carboxymethylenebutenolidase homolog (EC 3.1.-	0.004754	1.840435606	up
48	Ankyrin-3 (ANK-3) (Ankyrin-G)	0.002376	1.840435606	up
49	Translocase of outer mitochondrial membrane 20	0.016677	1.795062878	up

50	Vitronectin	0.001945	1.79246161	up
51	Pyridoxine 5'-phosphate oxidase variant (Fragmen	0.034765	1.791996277	up
52	CD166 antigen (Activated leukocyte cell adhesion	0.011752	1.779527559	up
53	Ferritin	0.019354	1.776492365	up
54	Glycerol-3-phosphate dehydrogenase [NAD(+)] (E(	0.00549	1.773925104	up
55	Aldo-keto reductase family 1 member B10 (EC 1.1	0.013064	1.773925104	up
56	Uncharacterized protein	0.001964	1.772181146	up
57	Apolipoprotein C-III (Apolipoprotein C-III variant 1	0.007528	1.770900693	up
58	Protein LOC102724159	0.000639	1.764976959	up
59	-	0.001216	1.748969308	up
60	Short-chain dehydrogenase/reductase 3 (EC 1.1.1	0.015429	1.739726027	up
61	cDNA FLJ59619, highly similar to Epoxide hydrolas	0.033507	1.735522116	up
62	cDNA FLJ51770, highly similar to Microsomal trigl	1.53E-05	1.730996814	up
63	rRNA methyltransferase 3, mitochondrial (EC 2.1.1	0.000549	1.730541648	up
64	cDNA FLJ76254, highly similar to Homo sapiens ga	0.006824	1.728058208	up
65	Lectin galactoside-binding soluble 3 binding prote	0.005207	1.721088435	up
66	Galectin-2 (Gal-2) (Beta-galactoside-binding lectin	0.000877	1.705139766	up
67	Arylacetamide deacetylase (EC 3.1.1.3)	0.000454	1.704688909	up
68	Sulfotransferase (EC 2.8.2.-)	0.001124	1.702702703	up
69	RAS p21 protein activator 4	3.21E-05	1.697841727	up
70	cDNA FLJ76489, highly similar to Homo sapiens so	0.010826	1.696629213	up
71	Lysosome-associated membrane glycoprotein 2 (L	0.001592	1.692998205	up
72	Alpha-fetoprotein	0.000777	1.688620072	up
73	Histamine N-methyltransferase (HMT) (EC 2.1.1.8)	0.000624	1.687724014	up
74	cDNA FLJ35730 fis, clone TESTI2003131, highly sin	0.004943	1.686968204	up
75	Redox-regulatory protein FAM213A (Peroxiredoxi	3.94E-05	1.685765443	up
76	cDNA FLJ55760, highly similar to Squalene synthet	3.03E-06	1.685317816	up
77	Transcriptional enhancer factor TEF-1 (Fragment)	0.0069	1.678125	up
78	Succinate dehydrogenase [ubiquinone] flavoprote	0.000544	1.670970614	up
79	PHD finger protein 6	0.002627	1.670226969	up
80	Ribonucleoside-diphosphate reductase large subu	0.000159	1.659131206	up
81	Membrane alanine aminopeptidase variant (Fragn	0.00047	1.656042497	up
82	MARCKS-related protein (MARCKS-like protein 1)	0.014895	1.651347768	up
83	NADH dehydrogenase [ubiquinone] 1 alpha subco	0.000503	1.648565121	up
84	Plasma membrane citrate carrier	0.003358	1.64084507	up
85	Galactosylceramidase, isoform CRA_c	0.000475	1.631140351	up
86	Epididymis tissue sperm binding protein Li 18mP (	0.002831	1.622377622	up
87	Zinc-alpha-2-glycoprotein (Zn-alpha-2-GP) (Zn-alpl	0.014558	1.610966057	up
88	Carbonic anhydrase 13 (EC 4.2.1.1) (Carbonate de	0.000849	1.609830361	up
89	E-cadherin 1	0.000176	1.605731654	up
90	Apolipoprotein A-IV (Apo-AIV) (ApoA-IV) (Apolipo	5.25E-06	1.605297438	up
91	Carboxypeptidase (EC 3.4.16.-)	0.000249	1.603732639	up
92	Erlin-2 (Endoplasmic reticulum lipid raft-associate	0.000937	1.603470716	up
93	Mitochondrial amidoxime-reducing component 1	0.024246	1.601908066	up
94	Protein NipSnap homolog 1 (NipSnap1)	0.001356	1.597402597	up
95	Fructosamine-3-kinase (EC 2.7.1.-)	0.00318	1.597402597	up
96	Carboxylic ester hydrolase (EC 3.1.1.-)	0.000919	1.595155709	up
97	Centromere protein V (CENP-V) (Nuclear protein p	0.003143	1.594723183	up
98	Prolyl 4-hydroxylase subunit alpha-2 (4-PH alpha-2	0.004179	1.592912705	up
99	cDNA, FLJ95508, highly similar to Homo sapiens 5	0.002504	1.590673575	up

100	Phospholipid phosphatase 3 (EC 3.1.3.4) (Lipid pho	0.029253	1.579535684	up
101	-	0.007341	1.579105761	up
102	cDNA, FLJ93317, highly similar to Homo sapiens ca	0.000167	1.578178694	up
103	Sulfotransferase (EC 2.8.2.-)	0.009039	1.575783598	up
104	28S ribosomal protein S9, mitochondrial (MRP-S9)	0.000178	1.574002574	up
105	Pyroglutamyl-peptidase 1 (EC 3.4.19.3) (5-oxoprol	0.00263	1.573573574	up
106	Protein FAM162A (E2-induced gene 5 protein) (Gr	6.18E-05	1.570694087	up
107	Gamma-aminobutyric acid receptor-associated pr	0.000468	1.569164882	up
108	Long-chain-fatty-acid--CoA ligase 5 (EC 6.2.1.3) (Lo	5.07E-05	1.565626336	up
109	Dimethylaniline monooxygenase [N-oxide-forming	0.001227	1.56477127	up
110	Cystatin	6.04E-05	1.560153584	up
111	Calcium binding protein P22, isoform CRA_a	0.000637	1.556881125	up
112	Methyltransferase like 7A, isoform CRA_a	0.000191	1.553191489	up
113	Apolipoprotein B (Including Ag(X) antigen) (Apolip	0.002139	1.549936252	up
114	cDNA, FLJ95623, highly similar to Homo sapiens d	0.002992	1.546689304	up
115	Thioredoxin-dependent peroxide reductase, mitoc	0.001538	1.541949153	up
116	Glutathione peroxidase	0.000501	1.54171961	up
117	cDNA FLJ10554 fis, clone NT2RP2002385, highly si	0.015616	1.539145154	up
118	Protein disulfide-isomerase A5 (EC 5.3.4.1) (Protei	0.00022	1.538071066	up
119	Epididymis secretory protein Li 41	0.008101	1.538071066	up
120	cDNA FLJ76718	0.002942	1.532714225	up
121	Succinate--CoA ligase [GDP-forming] subunit beta,	0.000303	1.526315789	up
122	Gamma-glutamyl carboxylase (Gamma-glutymyl c	0.010209	1.522068096	up
123	SPATS2-like protein (DNA polymerase-transactivat	0.004141	1.518471872	up
124	Prothrombin (EC 3.4.21.5) (Coagulation factor II) [	0.002071	1.51572327	up
125	Fructose-bisphosphate aldolase (EC 4.1.2.13)	0.004158	1.514034353	up
126	4-aminobutyrate aminotransferase, mitochondria	0.000342	1.513196481	up
127	Tudor and KH domain-containing protein	0.000997	1.511511093	up
128	Carboxypeptidase (EC 3.4.16.-)	0.013298	1.510460251	up
129	28S ribosomal protein S35, mitochondrial (MRP-S3	0.001003	1.510460251	up
130	Reelin	0.024128	1.508779264	up
131	Microsomal glutathione S-transferase 1 (Microsor	0.000791	1.508361204	up
132	Zinc finger RNA binding protein	0.027207	1.506683375	up
133	Carnitine O-acetyltransferase (Carnitine acetylase	0.001339	1.506683375	up
134	Acyl-coenzyme A thioesterase 13 (Acyl-CoA thioes	0.001465	1.506683375	up
135	REST corepressor 3	0.013482	1.503128911	up
136	Peptidylprolyl isomerase (EC 5.2.1.8)	0.002564	1.502502085	up
137	Putative uncharacterized protein DKFZp686B0215	0.003862	1.502085071	up
138	Epididymal secretory protein E1 (Fragment)	0.000351	1.501042101	up
139	Receptor protein-tyrosine kinase (EC 2.7.10.1)	0.001377	0.663893511	down
140	Actin, alpha cardiac muscle 1 (Alpha-cardiac actin)	0.000515	0.663432215	down
141	Heat shock 70 kDa protein 1B	0.000547	0.659933592	down
142	Annexin	8.04E-05	0.659568584	down
143	PRKR interacting protein 1 (IL11 inducible), isofor	0.021589	0.657640232	down
144	Cytochrome c oxidase subunit 7C, mitochondrial (	0.032494	0.652892562	down
145	Aldo-keto reductase family 1 member C3	0.000491	0.652257709	down
146	Filamin-B (FLN-B) (ABP-278) (ABP-280 homolog) (A	9.58E-06	0.650343879	down
147	cDNA FLJ59614, highly similar to Ubiquitin fusion	0.016999	0.647446458	down
148	Lymphocyte cytosolic protein 1 (L-plastin), isoforn	0.000187	0.646994236	down
149	Secernin 1	0.002544	0.645462024	down

150	High mobility group AT-hook 1	0.003785	0.645010965	down
151	Uncharacterized protein (Fragment)	0.010586	0.644560153	down
152	HSPA8 protein (Fragment)	0.011805	0.644560153	down
153	14-3-3 protein sigma (Epithelial cell marker protei	0.00017	0.643835616	down
154	Gelsolin (AGEL) (Actin-depolymerizing factor) (ADI	8.15E-05	0.643659271	down
155	Epididymis secretory protein Li 22 (Glutathione S-	0.000341	0.636388434	down
156	Radixin (Radixin, isoform CRA_b) (cDNA, FLJ93349	0.001558	0.62999185	down
157	Poliovirus receptor (Poliovirus receptor, isoform C	0.001502	0.624593716	down
158	Calponin	0.001424	0.623545332	down
159	Glycerophosphodiester phosphodiesterase domai	0.001932	0.622498648	down
160	Annexin	0.000119	0.621891892	down
161	MHC class II regulatory factor RFX1 (Enhancer fact	0.013333	0.61987041	down
162	Neuroblast differentiation-associated protein AHN	9.42E-06	0.616814875	down
163	Cystathionine gamma-lyase (EC 4.4.1.1) (Cysteine-	0.000215	0.61637931	down
164	Protein S100-A4 (Calvasculin) (Metastasin) (Placer	0.000138	0.613336919	down
165	Ras-related protein R-Ras2 (Ras-like protein TC21)	3.48E-07	0.611006711	down
166	Glycine amidinotransferase (L-arginine:glycine am	0.000956	0.609010459	down
167	Cathepsin E (EC 3.4.23.34) [Cleaved into: Cathepsi	0.00354	0.607449089	down
168	Transgelin-2 (Epididymis tissue protein Li 7e) (SM2	0.007911	0.604707141	down
169	Testicular secretory protein Li 7	5.96E-05	0.60042678	down
170	Aldo-keto reductase family 1 member C1 (EC 1.1.1	0.000276	0.593891102	down
171	Myosin-9 (Cellular myosin heavy chain, type A) (M	0.00056	0.59009009	down
172	Proteasome-associated protein ECM29 homolog	0.000957	0.588141874	down
173	Gap junction protein	0.000486	0.587566138	down
174	Aldose reductase (AR) (EC 1.1.1.21) (Aldehyde red	0.000167	0.582124967	down
175	Protein CDV3 homolog	0.00028	0.580874605	down
176	Actin-like protein (Fragment)	5.16E-05	0.58061117	down
177	Nucleotide triphosphate diphosphatase NUDT15 (	0.000681	0.579515534	down
178	cDNA FLJ39529 fis, clone PUAEN2004067, highly s	0.004109	0.57480315	down
179	4F2 cell-surface antigen heavy chain (Solute carrie	4.18E-06	0.573714586	down
180	Switch-associated protein 70 (SWAP-70)	3.72E-05	0.573301862	down
181	High mobility group protein HMG-I/HMG-Y (HMG-	0.004642	0.567398119	down
182	Clathrin light chain	7.73E-05	0.567398119	down
183	Filamin-A (FLN-A) (Actin-binding protein 280) (ABF	5.90E-06	0.561540463	down
184	Neutral amino acid transporter B(0) (ATB(0)) (Bab	3.26E-05	0.560728218	down
185	Lactoferrin	0.000988	0.547447138	down
186	Claudin-6 (Skullin)	0.002914	0.546391753	down
187	S100 calcium binding protein A10 (Annexin II ligan	0.000648	0.543209877	down
188	Large neutral amino acids transporter small subur	0.000274	0.542813062	down
189	15-hydroxyprostaglandin dehydrogenase [NAD(+)]	0.000485	0.542673522	down
190	cDNA, FLJ92068, highly similar to Homo sapiens R	0.001377	0.537416709	down
191	ENO2 protein (Enolase 2 (Gamma, neuronal), isofo	0.003453	0.527629234	down
192	Serpin B5 (Maspin) (Peptidase inhibitor 5) (PI-5)	4.19E-05	0.517834556	down
193	Zinc finger protein 106 homolog (Mouse), isoform	0.001738	0.516683519	down
194	Tumor-associated calcium signal transducer 2 (Cel	0.037021	0.506402209	down
195	Glutathione S-transferase P (Fragment)	0.000375	0.491053678	down
196	Oligoribonuclease, mitochondrial (EC 3.1.-.-) (RNA	0.000541	0.488343254	down
197	Protein S100-A6 (Calcyclin) (Growth factor-induci	0.002203	0.469147894	down
198	Aldo-keto reductase family 1 member C2 (EC 1.-.-.	4.51E-05	0.464128843	down
199	Annexin A3 (35-alpha calcimedin) (Annexin III) (An	9.64E-05	0.461632156	down

200	Protein S100 (S100 calcium-binding protein)	0.005347	0.457371873	down
201	Keratin, type II cytoskeletal 7 (Cytokeratin-7) (CK-7	0.002566	0.454192923	down
202	Tropomyosin alpha-4 chain (TM30p1) (Tropomyos	3.45E-05	0.451488023	down
203	Transgelin	1.75E-06	0.450435203	down
204	TMSB4X protein (Fragment)	0.002571	0.42551675	down
205	Coactosin-like protein	9.97E-06	0.424400665	down
206	HMG domain-containing protein 3 (HMG box-cont	0.001544	0.409774436	down
207	Protein S100-P (Migration-inducing gene 9 protein	0.000141	0.398927489	down
208	Alpha-2-macroglobulin (Alpha-2-M) (C3 and PZP-li	0.000136	0.385361348	down
209	Protein S100-A5 (Protein S-100D) (S100 calcium-b	0.000598	0.357546956	down
210	Histone H1.1 (Histone H1a)	8.55E-06	0.327727373	down
211	Mutant hemoglobin alpha 2 globin chain	0.000901	0.319261214	down
212	cDNA, FLJ93654, highly similar to Homo sapiens se	0.006453	0.289490651	down

Antibody	Brand	Serial Number	Dilution
Anti-SCD1	Abcam	Ab19862	1:100
Anti-YAP	CST	#14074S	1:200
Anti-TEAD1	Abcam	Ab133533	1:100
Anti-Cyclin D1	CST	#55506	1:100
Anti-histone H3	CST	#4499S	1:200
Anti-β-actin	CST	#8457S	1:400
Anti-Metallothinein	Abcam	Ab12228	1:50
Anti-Transthyrein	CST	#29872S	1:400
Anti-Vitamin D binding protein	Abcam	Ab81307	1:200
Anti-Mikine	Abcam	Ab52637	1:200

Table S2. Detailed information of antibodies applied in western blotting.

Gene Name	Forward	Reverse
SCD1	5’ -TACTTGGAAGACGACATTCGC	5’ -GGTGTAGAACTTGCAGGTAGGA
Sox-2	5’- GAGGGCTGGACTGCGAACT	5’ -TTTGCACCCCTCCCAATTC
Oct-4	5’ -TGGGATATACACAGGCCGATG	5’-TCCTCCACCCACTTCTGCAG
Nanog	5’-ACCCAGCTGTGTGTACTCAA	5'-GGAAGAGTAAAGGCTGGGGT
β-actin	5’-GCCGGGACCTGACTGACT	5’-TGGTGATGACCTGGCCGT

Table S3. Primer sequences applied in real-time RT-PCR.

No.	Name	Target Sequence	CDS	GC%
SCD1-KD1	SCD-RNAi(70863-1)	cgTCCTTATGACAAGAACATT	491..1570	31.58%
SCD1-KD2	SCD-RNAi(70864-1)	ctACGGCTCTTTCTGATCATT	491..1570	42.11%
SCD1-KD3	SCD-RNAi(70865-1)	ccCACCTACAAGGATAAGGAA	491..1570	42.11%

Table S4. Primer sequences applied in the construction of lentivirus-siRNA.

Variables	SCD1 high	SCD1 low	*P* value
**Gender（M/F）**	34/21	21/17	0.527
**Age(＞50/＜50）**	35/20	25/13	0.831
**Tumor size (mm)**	30.5±14.3	28.4±12.1	0.220
**Lauren Classification**			0.020
**Intestinal**	45	15	
**Diffuse**	17	16	
**pTNM stage**			**＜0.001**
**I**	13	6	
**II**	11	15	
**III**	22	5	
**IV**	17	4	
**Lymph node metastasis （P/N）**	43/11	29/9	0.704
**Distant metastasis （P/N）**	12/43	2/36	**0.038**

Table S5. Clinico-pathological variables and the expression of SCD1 in PLAGH gastric cancer database

M, male; *F*, female; *P*, positive; *N*, negative.
